# Which psychosocial job demands matter most for parasympathetic heart rate variability? A dominance analysis study

**DOI:** 10.1093/joccuh/uiag025

**Published:** 2026-04-22

**Authors:** Kati Karhula, Maria Hirvonen, Hanna Jantunen, Maria Sihvola, Jarno Turunen, Piia Seppälä

**Affiliations:** Finnish Institute of Occupational Health, Comprehensive Security in Working Life, Panuntie 6, 00620 Helsinki, Finland; Finnish Institute of Occupational Health, ICT and Digital Services, Neulaniementie 4, 70210 Kuopio, Finland; Finnish Institute of Occupational Health, Occupational Health, Panuntie 6, 00620 Helsinki, Finland; Finnish Institute of Occupational Health, Comprehensive Security in Working Life, Panuntie 6, 00620 Helsinki, Finland; Finnish Institute of Occupational Health, ICT and Digital Services, Hoitajanrinne 1, 90220 Oulu, Finland; Finnish Institute of Occupational Health, Work Ability and Working Careers, Arinatie 3, 00370 Helsinki, Finland

**Keywords:** autonomous nervous system, psychophysiology, working conditions, public sector, heart rate variability

## Abstract

**Objectives:**

Prolonged reduced heart rate variability (HRV) is associated with impaired health and chronic diseases. Despite this connection, it remains unclear which psychosocial job demands are the strongest predictors of reduced HRV and poorer health outcomes. This study aimed to determine the most relevant psychosocial job demands that predict reduced HRV.

**Methods:**

The study participants were 163 municipal employees (86% female, mean age 47) who responded to a survey on psychosocial job demands and measured their electrocardiography-based HRV over 4 consecutive nights during a normal work week. The root mean square of successive RR intervals (RMSSD) was used as an indicator of autonomic nervous system activity mediated by the vagus nerve. Hierarchical mixed model regression analysis included psychosocial job demands and the most relevant individual factors and occupational sector as a nested random effect. Dominance analysis (DA) was used to assess all the variable combinations to identify the most significant determinants of HRV across the regression models.

**Results:**

The participants’ RMSSD was stable over the measurement period. DA ranked the participants’ age as the factor that most affected RMSSD. The psychosocial job demands that seemed to be the most relevant for RMSSD were encountering bullying and violence at work, ethically challenging situations at work, and effort-reward imbalance. Gender was ranked as the fourth factor.

**Conclusions:**

These results need to be confirmed in further studies, but they suggest that workplace bullying and violence as well as ethically challenging situations at work might have the greatest effect on HRV among public sector employees.

## Teaser

**Key points**
 

- **What is already known on this topic:** Different psychosocial job demands are related to an imbalance in the autonomic nervous system during which sympathetic activity continuously dominates. When prolonged, this state is associated with an increased risk of impaired health and several chronic diseases.
- **What this study adds:** However, which psychosocial job demands are the most relevant predictors of poorer health is still unknown. In line with previous studies, the dominance analysis identified age as the factor that most affected heart rate variability. The most relevant psychosocial demands were encountering bullying and violence at work, ethically challenging situations at work, and effort-reward imbalance.
- **How this study might affect research, practice or policy:** Future studies on the associations between the combined effects of different job demands and HRV are warranted. Research weighing the most important job demands and how they balance out the job resources for good HRV would be beneficial.

## 1. Introduction

Autonomic nervous system (ANS) imbalance refers to a situation when sympathetic nervous system (SNS) activity continuously dominates parasympathetic nervous system (PNS) activity.^1^ Prolonged autonomic imbalance, manifested as reduced heart rate variability (HRV), is associated with impaired health and increased risk of different chronic diseases,^2^ such as the progression of coronary atherosclerosis^3^ and even cardiovascular morbidity.^4^^,^^5^

ANS activity is noninvasively assessed by measuring HRV, which is mainly controlled by the ANS.^6^ A higher percentage of heart rate reserve while working is associated with lower HRV indices the following night, which reflects an imbalance in autonomic cardiac modulation.^7^ Conversely, high physical load can lead to attenuated parasympathetic cardiac modulation.^7^

Measuring HRV during sleep is a reliable way to determine HRV and interpret PNS activity, as it does not require time and effort in measuring different work and leisure time physical activities or controlling the various environmental factors, such as changes in temperature and noise levels, that are present when measurements are taken while awake.^8–11^ Of the different time-domain indices, RMSSD (root mean square of successive RR intervals) is often regarded as the most relevant.^12^^,^^13^ Whereas both high-frequency (HF) power and RMSSD are parameters that reflect parasympathetic activity, HF is strongly dependent on breathing (both the rhythm and depth of breathing affect its value).^11^ As RMSSD does not depend on the respiratory frequency it is often preferred to measure parasympathetic activity.^11^ The average HRV of the whole night is used, as this reduces HRV difference in different sleep stages.^9^

Psychosocial job demands include various factors such as excessive workload,^14^ conflicting expectations at work,^15^ and qualitative job demands, such as complex decision-making.^16^ Which psychosocial job demands are the most relevant predictors of poorer health is still not known. However, consistent research evidence shows that psychosocial job demands are related to an increased ANS imbalance.^17^ So far, studies utilizing work stress surveys have generally found an association between stress and lower HRV.^17–19^ In cases of single specified job demands, interpersonal conflicts in the workplace have been associated with lower night-time HRV during the following night.^20^ Higher quantitative work demands were associated with poorer recovery during the working day, shown by HRV.^21^

However, to the best of our knowledge, no previous study has investigated the relative importance of various job demands for parasympathetic HRV. To fill this research gap, the aim of this study was to determine the main psychosocial job demands that influence night-time parasympathetic HRV during the working week, and which job demands predict decreased HRV.

## 2. Methods

### 2.1 Study population and design

This study was conducted among a sample (*n* = 240) of Finnish municipal employees (*n* = 1083) recruited from volunteered workplaces in daycare, expert or administrative work, food services, and managerial positions, including the aforementioned sectors. Each voluntary participant (*n* = 198, participation rate 82.5%) completed an electronic questionnaire, which measured job demands, demographic variables, and background variables such as medical illness.

Two research nurses visited the workplaces to guide and instruct the participants in measuring their HRV, monitoring their ambulatory activity, and filling in a research diary. The participants received detailed oral and written instructions for successfully following the recording protocol. They could also contact the research nurse during office hours if they had any problems. The participants were instructed to maintain their normal daily routines, but to avoid consuming alcohol during the HRV measurements. During the ambulatory monitoring, the participants kept a diary describing their daily activities and noting any factors that could potentially affect their HRV measurements (eg, self-reported sleep, medication, acute sickness, and alcohol consumption).

To reduce bias in the HRV measurements, the following medical conditions and/or medications were used as exclusion criteria: heart pacemaker, heart transplant, severe heart disease, persistent atrial fibrillation or atrial flutter, uncontrollable thyroid dysfunction, and alpha- or beta-receptor blockers. Being pregnant or having severe obesity, that is, a body mass index (BMI) >40 kg/m^2^,^11^ were also exclusion criteria.

**Figure 1:**
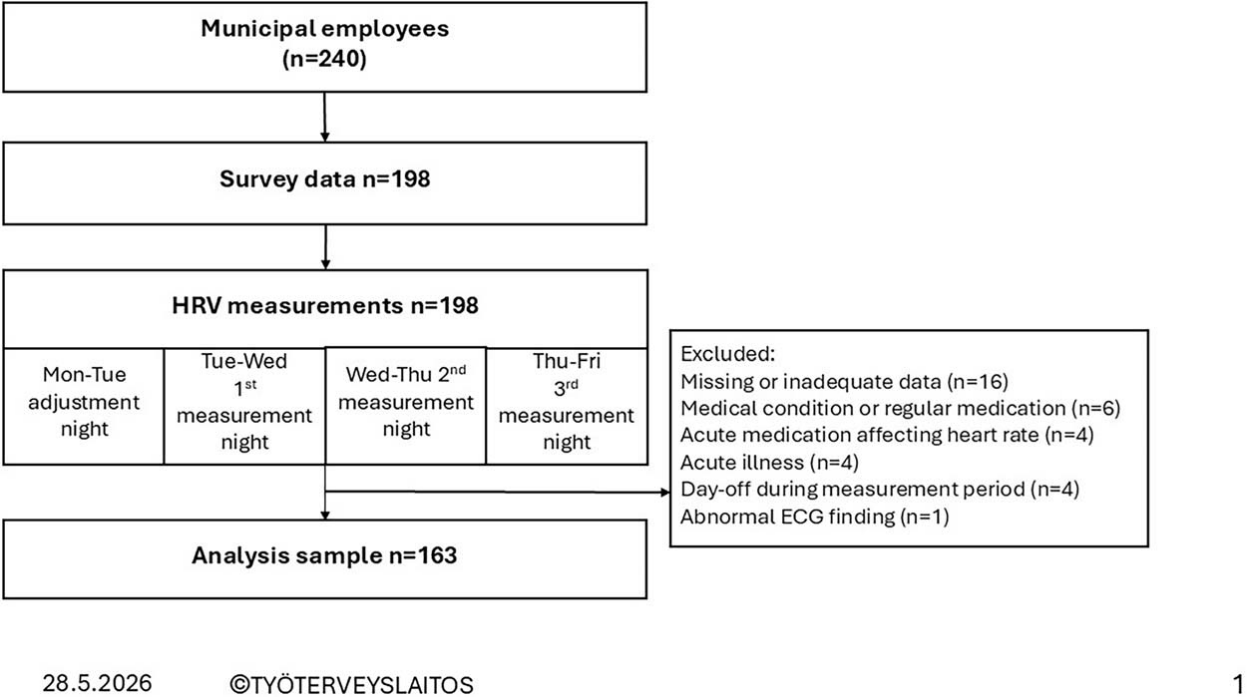
Study flow chart.

HRV was measured using a Bittium Faros™ 360 ambulatory device (Bittium Medical Technologies, Oulu, Finland). Single-channel electrocardiography (ECG) was used to measure heartbeat-to-heartbeat intervals (RRIs). These RRIs were recorded for 4 consecutive nights. The Faros device was attached to the chest at bedtime and removed in the morning upon waking. The participants marked their bedtime and wake-up times in the diary, and these times were stamped to the HRV raw data. The mean times for going to bed and waking up were 09:41 pm and 06:15 am for the second night, 09:52 pm and 06:14 am for the third night, and 08:45 pm and 06:11 am for the fourth night. As the Faros device has an acceleration sensor (accelerometer) and a gyroscope, the motion data could also be used to determine when a person is in an upright position in case of any uncertainties. The first measurement night (Monday/Tuesday) was an adjustment night for familiarization with the electrodes and was therefore excluded from the analyses to improve the accuracy of the measurements. The 3 subsequent nights (ie, Tuesday to Friday) were included in the analysis to mitigate the impact of transient changes and provide a more reliable HRV measure. Figure 1 presents the study design.

The participants worked morning shifts (scheduled between 06:00 am and 09:30 pm, starting before midday) and afternoon shifts (scheduled between midday and 10:00 pm, starting at or after midday). None of the participants worked night shifts during the measurement period. The measurements were conducted between September and early December 2023. On a weekly basis, approximately 40 employees responded to the questionnaire and measured their HRV.

### 2.2 Measures

#### 2.2.1 Psychosocial job demands

The participants rated 5 statements on a 5-point scale ranging from “strongly agree” to “strongly disagree” to explore *quantitative job demands*. An example is “I don't have enough time to get my work done”.^14^  *Qualitative job demands* were assessed on the basis of responses to 6 questions from the QPS Nordic survey (Nordic General Survey of Psychological and Social Factors at Work): for example, “Does your work require complex decisions?” and “Does your job require that you acquire new knowledge and new skills?” The participants also rated these on a 5-point scale ranging from “very rarely or never” to “very often or always.”^16^

*Conflicting expectations at work* were elicited by 1 question: “How often in the last six months have you been clearly disturbed, worried or stressed by different and conflicting expectations of your job from different sources?” to which the participants responded using a 5-point scale ranging from “very rarely or never” to “very often or constantly.”^22^ Responses to the item “In *ethically challenging situations*, people are forced to reflect on the rightness and goodness of one’s actions, choices and decisions, and it is not always clear what would be the right thing to do. How often do you face ethically challenging situations at your work?" were assessed using a 5-point scale ranging from “never” to “almost every day.”^23^ Social isolation was assessed by 4 statements, such as “I am isolated from others at work,” using a 7-point scale of responses ranging from “strongly disagree” to “strongly agree.”^24^

The 3 statements on *hindrance job demands* were: “The rules and regulations of my workplace prevent me from carrying out my work properly,” “It often takes too long to get the tools or information that I need,” and “Decisions concerning my work are made by people who do not understand my work.” Responses were rated on a 5-point scale ranging from “very rarely or never” to “very often or constantly,”^25^ adopted from Schaufeli.^26^ Work underload was elicited by the statements “I don't have enough to do at work” and “My work tasks are not challenging enough,” the responses to which were on a 5-point scale ranging from “very rarely” to “very often.”^27^

Altogether 19 statements were used to examine *work intensification* (work intensification, job-related planning and decision-making demands, career-related planning and decision-making demands, knowledge-related learning demands, and skill-related learning demands) over the preceding year. Examples are “I have more often had to make decisions at work without consultation with supervisors” and “I have increasingly had to do two or three things at once,” which were rated on a 5-point scale ranging from “not true at all” to “absolutely true.”^28^ The *mismatch between rewards and effort at work* was elicited by the statement “When I compare the effort I put into my work (eg, time, energy, effort) with the rewards I receive (eg, salary, status, recognition), in return I get...,” which had 5 response options ranging from “much less than I put in” to “much more than I put in.”^29^

*Workplace bullying and violence* during the preceding 3 months was elicited by 4 yes/no statements. We also asked about difficult customer and interaction situations with a dichotomous question: “Does your work involve a lot of difficult customer and interaction situations that arouse negative feelings?” which had dichotomous response options.

#### 2.2.2 Heart rate variability

Kubios HRV Scientific (version 4.0.3) (Kubios Ltd, Kuopio, Finland) was used to analyze the RRI data and to scan and correct any artefacts. If the value of the corrected beats was 5% or more, or the measurement period contained 20% or more corrected artefacts, the measurement period was excluded from further analyses.^30^ A single time-domain measure of RMSSD was used and was computed on the basis of the variability in the intervals between successive RRIs, in line with the Task Force Guidelines.^12^ After this, we aggregated the measurement period by computing the RMSSD mean values (ms) for the 3-night period under study. We then aggregated the measuring period by computing the mean RMSSD values to reduce any unpredictable confounding influences.^31^

#### 2.2.3 Confounding variables

Age, gender, BMI, caffeine and nicotine consumption, symptoms or diseases not in the exclusion criteria (eg hypertension, hypo- or hyperthyroidism), and the use of alcohol or medication not in the exclusion criteria were used as control variables in the statistical analyses because of their influence on HRV.^12^^,^^13^^,^^32^ The participants were instructed not to consume any alcohol during the HRV measurement period. However, if they did report alcohol consumption (*n* = 5, consumption of 1 or 2 doses), this was included as a control variable.

### 2.3 Ethical issues

The study protocol was accepted, and ethical approval was obtained from the Ethics Board (HUS/2825/2023) before the recruitment of the participants. The study procedures were in accordance with the ethical standards of the Helsinki Declaration. All the participants were voluntary and gave their informed consent. The participants did not receive any monetary compensation for their contribution. The employer was not aware which of the invited employees participated in the study, and neither participation nor nonparticipation affected the treatment of employees in the workplace.

All the participants were sent personal feedback on their measurement results via secured email. The doctor in charge of the study contacted the participants if there were any abnormalities in their HRV measurements. All the participants could contact the doctor in charge of the study to discuss their feedback.

### 2.4 Statistical analysis

The statistical analyses used hierarchical mixed model regression and dominance analysis (DA) to examine the relative importance of the predictors in terms of their proportional contribution to the explained variance in RMSSD.^33^ Among its applications, DA is used to overcome methodological issues in traditional regression models that have several correlated predictors.^34^ The analysis compared the impact of different job demands on HRV with each other, to identify the most important factors. The analysis began by examining the effect of a single job demand, and the variables in the model were increased until the strongest predictors were identified. Occupational sector was added to the model as a nested random effect. To improve the overall fit of the model, the final model includes the 5 background variables ranked as the most important in the broader model that included age, BMI, caffeine and nicotine consumption, physical activity, medication, illness, and alcohol consumption (results not reported here). The analysis was conducted using STATA 18.0 (StataCorp LLC, TX, USA).

## 3. Results

### 3.1 Final sample and descriptive characteristics

Of the 198 starting the HRV measurements, a total of 35 participants were excluded from the final sample for the following reasons: 6 participants had a physiological condition or disease leading to exclusion; 4 had medication for an acute condition that was different from their regular medication and affected their heart rate; 4 had a day off or retirement day during the measurement period; 4 became acutely ill during the measurements; and 1 had an abnormal electrocardiogram. An additional 16 measurements were excluded due to insufficient HRV data, 8 of which did not meet the predefined criteria for data quality and 8 of which had missing data from at least 1 measurement night.

Of the participants in the analysis sample (*n* = 163) 86% were female, with a mean age of 47 years. They were most often employed in daycare (40%) and expert or administrative work (32%). Smaller proportions of participants worked in food services or had a managerial position in the aforementioned occupational sectors (Table 1).

**Table 1:** Characteristics of study participants (*n* = 163).

Characteristics	Mean	(SD)	Min to max
**Age, y**	46.53	(11.57)	22-65
**Body mass index** ^**a**^	26.33	(4.43)	17.26-38.30
**Work experience, y**	22.85	(10.87)	1-47
**Sleep duration (h:min)**	7:36	(0:42)	5:12-9:42
**Working time** ^**b**^ **(h:min)**	7:57	(0:44)	4:10-10:40
	**%**	**(*n*)**	
**Gender**			
**Female**	86.5	(141)	
**Male**	13.5	(22)	
**Type of employment contract**			
**Permanent**	95.7	(156)	
**Fixed-term**	4.3	(7)	
**Working hours**			
**Full-time**	96.9	(158)	
**Part-time**	3.1	(5)	
**Employment in:**			
**Daycare**	39.9	(65)	
**Expert or administrative work**	33.7	(55)	
**Food services**	11.7	(19)	
**Managerial position** ^**c**^	14.7	(24)	
**Supervisory tasks**			
**Yes**	21.5	(35)	
**No**	78.5	(128)	
**Stimulant intake**			
**Caffeine (yes)**	93.3	(152)	
**Nicotine (yes)**	7.4	(12)	
**Alcohol consumption** ^**b**^ **(yes)**	3.1	(5)	

^a^Self-reported, weight (kg)/height (m)^2^.

^b^During heart rate variability measurement period.

^c^Includes managers in daycare and food services.

Approximately three-quarters of the participants rated their workability as at least good (76%), and two-thirds rated their health as at least good (67%). The 3 working days included in the analysis consisted of, on average, a little under 8 hours of working time and an average of slightly over 7½ hours of sleep (Table 1). The night-time HRV measurements on the 3 measurement nights showed an average heart rate of 61 bpm and an average RMSSD of 41.

### 3.2 Results from the DA

DA identified the participants’ age as the factor that most affected RMSSD in hierarchical mixed model regression models. The psychosocial job demands that might be most relevant to RMSSD were encountering workplace bullying and violence at work, and ethically challenging situations at work. Gender was ranked as the fourth most important factor, and effort-reward imbalance as the fifth (Table 2.)

**Table 2:** Results of dominance analysis.

Job demands and background variables	Ranking	Standardized dominance statistics ^b^	Standardized beta ^c^
**Age, y** ^**a**^	1	0.6609	−.47724^***^
**Workplace bullying and violence**	2	0.3169	.13830
**Ethically challenging situations**	3	0.1944	.19189^*^
**Gender** ^**a**^	4	0.1000	−.07509
**Effort-reward imbalance**	5	0.0966	−.09010
**Social isolation**	6	0.0537	.01514
**Difficult customer and interaction situations**	7	0.0219	−.16954
**Disease** ^**a**^	8	0.0174	−.35467^**^
**Hindrance job demands**	9	0.0161	−.09945
**Body mass index** ^**a**^	10	0.0141	.06776
**Conflicting expectations at work**	11	−0.0104	.13394
**Work intensification**	12	−0.0274	−.09499
**Medication** ^**a**^	13	−0.0400	.34732^**^
**Quantitative job demands**	14	−0.0501	−.02045
**Qualitative job demands**	15	−0.1365	−.09685
**Work underload**	16	−0.2276	.02649

^a^The model includes the 5 most relevant background variables.

^b^Standardized dominance statistics do not total to 1 due to rounding.

^c^^*^ *P* ≤ .05; ^**^P ≤ .01; ^***^P < .001.

The overall explanatory power of the model was 0.5283. Notably, conflicting expectations at work, job intensification, prescription medication, quantitative and qualitative job demands, and job underload had a negative standardized dominance, which may indicate that these variables are influenced by or through other variables (multicollinearity or variable interactions).

In addition, in the regression model with the same variables, the strongest explanatory factors for HRV (with the highest beta values) were age, illness, and medication. Of the job demands, only ethically challenging situations had a statistical significance of ≤.05 (Table 2).

## 4. Discussion

The aim of this study was to investigate the main psychosocial job demands that influence night-time HRV during the working week, and which job demands predict reduced HRV (namely RMSSD) the most compared with the other job demands. Our main finding was that the participants’ age was the factor that most affected RMSSD. The psychosocial demands that might be most relevant to RMSSD were encountering workplace bullying and violence at work, and ethically challenging situations at work. Gender was ranked as the fourth most influential factor, and effort-reward imbalance as the fifth.

Previous studies^11^^,^^32^ have also found that age is a factor that greatly reduces HRV. Previous research has studied separate job demands, but the results of these studies cannot be compared with those of this study, which investigated the relative importance of various job demands in comparison with one another. There is evidence of an association between work stress measured by surveys in particular, and lower HRV,^17–19^ the job demands-control model^35^ and effort-reward Imbalance model being among the studied work stress models.^18^ To the best of our knowledge, no previous studies have investigated the association between workplace bullying and violence or ethically challenging situations at work and HRV during sleep.

Recovery during sleep is crucial for perceived recovery and stress resilience. Therefore, it is plausible to assume that, in these data, the good average self-assessed sleep duration of the participants contributed to the predominantly good measured HRV and perceived health estimations.^36^

Taking into account the length of the measurement period and the amount of psychophysiological data collected, one of the main strengths of this study was its relatively large sample. Previous studies have had at most, approximately half the number of participants of this study.^19^^,^^20^ Additionally, the over 80% participation rate can be regarded as good for a study that requires several days of commitment from the participants. In describing parasympathetic HRV, we utilized RMSSD, which is the most used time-domain metric for that purpose and recommended by the guidelines for measurement standards.^12^

Additionally, the employees selected for this study were from different occupational sectors and were comparable in terms of average age and the female dominance among public sector employees.^37^ Even though the participants were largely middle-aged females, both characteristics known to increase the risk of sleep disturbances,^38^ it has also been shown that fragmented sleep does not affect cardiac autonomic parameters.^39^

Using DA as a statistical method enabled the inclusion of variables measured on different scales in the statistical model.^33^ In addition, DA used the multivariate and linear mixed-effect model, which considered the participants' occupational sector as a (nested) random effect to account for the differences in work tasks and workload. For example, this acted as a way to control for the fact that physical job demands play a role in HRV.^7^ The results provide valuable comparisons in identifying the most promising predictors for potential future interventions.

Some limitations must also be addressed. The DA results are indicative and should be confirmed in future studies. The information on the participants’ health status and medication was based on self-reports, which are prone to inaccuracy and memory bias. The likelihood of this limitation was diminished by the specificity of the questions in the survey and by the participants keeping a diary over the HRV measurement period. The diary data were not comprehensive in terms of workload and specific peaks during the day, so it is possible that not all the factors that affected the next night's HRV were taken into account. However, using the average RMSSD for the 3 measurement nights means that possible individual peaks on individual days are not highlighted in the data. Additionally, the effects of stimulants and drugs, for example, were considered, and the study had rather strict exclusion criteria for health-related factors.

Future studies are warranted not only on the associations between the combined effects of different job demands, but also on the combined effects of certain job demands and HRV, such as ethically challenging situations at work, workplace bullying and violence at work, hindrance job demands, and work underload. An analysis that would weigh the most important job demands and how they balance out the job resources for good HRV would also be beneficial in this area of research.

## 5. Conclusions

The results of this study need to be confirmed in further research, but they suggest that workplace bullying and violence, and ethically challenging situations at work might be the most relevant factors affecting HRV among public sector employees.

## Data Availability

The research data are not available either publicly or on request from the authors or the Finnish Institute of Occupational Health due to privacy and ethical restrictions.
